# Analyzing the bibliometrics of brain-gut axis and Parkinson’s disease

**DOI:** 10.3389/fneur.2024.1343303

**Published:** 2024-03-07

**Authors:** Lingshan Chen, Jianfei Chen, Min Wu, Pingkang Yan, Xueping Zhou

**Affiliations:** ^1^Medical Laboratory Specialty, The Second Hospital of Jinhua, Jinhua, Zhejiang Province, China; ^2^School of Medical Technology and Information Engineering, Zhejiang Chinese Medical University, Hangzhou, China; ^3^Department of Gerontology, The Second Hospital of Jinhua, Jinhua, Zhejiang Province, China

**Keywords:** brain-gut axis, Parkinson’s disease, bibliometric analysis, Bibliometrix R, VOSviewer

## Abstract

**Background:**

Parkinson’s disease (PD), characterized by the loss of dopaminergic neurons, is a progressive neurodegenerative disorder. Recent research has revealed a significant connection between gut microbiota and PD. To gain insight into research interests, disciplinary contexts, and potential future directions, a comprehensive bibliometric analysis was conducted on the brain-gut axis and PD literature published between 2014 and 2023.

**Methods:**

Relevant literature records were gathered from the Web of Science Core Collection on August 11, 2023. The data were then analyzed by Biblioshiny R packages and VOSviewer (version 1.6.19).

**Results:**

The dataset revealed an upward trend in annual scientific publications on the brain-gut axis and PD, with an annual growth rate of 50.24%. China, the United States, and Italy were the top three most productive countries/regions. The journal “International Journal Of Molecular Sciences” published the most articles, while “Movement Disorders” received the highest number of citations. Professor Keshavarzian A emerged as the most prolific author, while Professor Scheperjans F held the highest h-index. Keyword analysis highlighted “alpha-synuclein” as the most frequent term, with “mouse model,” “inflammation,” and “risk” as emerging research topics. Additionally, “central nervous system” and “intestinal bacterial overgrowth” attracted increasing attention.

**Conclusion:**

This study examined current trends and hotspots in the bibliometric landscape of the brain-gut axis and PD research. Future research directions should explore the functional and metabolic activities of gut microbiota. Additionally, transitioning from observational to interventional study designs offers the potential for personalized interventions and disease prediction. These findings can guide researchers in navigating the latest developments and shaping the future directions of this field.

## Introduction

It is well-established that Parkinson’s disease (PD) is the second most common neurodegenerative disease, with an estimated prevalence of about 1–3% in people over the age of 65, and its prevalence increases with age (1). Due to population aging and environmental factors, PD has developed into one of the fastest-growing neurological diseases worldwide (2). Interestingly, recent studies have pointed to a potential connection between symbiotic gut bacteria and the brain that may impact neurodevelopment, brain function, and general health. The brain-gut axis is the term used to describe this two-way connection (3). A growing body of research suggests that the brain-gut axis may play a key role in the pathophysiology of Parkinson’s disease, given that non-motor symptoms, such as gastrointestinal manifestations, frequently appear before motor symptoms and the diagnosis of PD (4). However, there lacks a comprehensive and intuitive analysis of the current research hotspots and frontiers in the field of PD and brain-gut axis.

Bibliometric analysis provides a standardized methodology for extracting crucial information from relevant publications, such as main research themes, authors and countries covered, etc. (5). In this respect, bibliometric analysis can offer a perspective into the academic influence of an article by scrutinizing how many occasions it has been cited. Furthermore, by analyzing the frequency of keyword co-occurrence, researchers can gain insight into both established areas of focus and emerging trends within specific research domains (6).

Studies have reported consistent alterations in the composition of specific gut bacterial populations in individuals with Parkinson’s disease, regardless of their geographical location (7). Emerging evidence suggests that gut microbiota dysbiosis might play a role in the pathogenesis of PD by impacting various gut-related processes, including short-chain fatty acid (SCFA) production, lipid metabolism, immunological control, and intestinal permeability (7). Importantly, further research into the connection between PD and the brain-gut axis could promote the emergence of new diagnostic and treatment options for PD. Bibliometric analysis is a valuable tool in this context. The contributions and linkages between diverse study components are made more explicit, demonstrating the advancement of knowledge in the area. This study leverages bibliometric analysis, encompassing both performance evaluations and science mapping techniques, to comprehensively explore the existing research landscape on the brain-gut axis and its association with PD. Based on the analysis, the study aims to identify key research areas, performance trends, and emerging frontiers, yielding recommendations for future research directions.

## Materials and methods

### Bibliographical sources

The Web of Science Core Collection (WoSCC) is reportedly the most complete resource for science metric analysis (8). Relevant data were downloaded from the Science Citation Index Expanded (SCI-EXPANDED) in the WoSCC from Jan 01, 2014, to Jun 30, 2023. Keywords (Seen in Supplementary material 1) were searched using the title (*TI*), abstract (*AB*), and author keywords (*AK*) to obtain more accurate results as previously described (9, 10). Articles and Review articles were extracted and exported in either “Plain text file” or “Tab-delimited file” formats. The data were recorded as “Full record and cited references” to ensure comprehensive coverage (11).

### Data analysis

**Figure 1:**
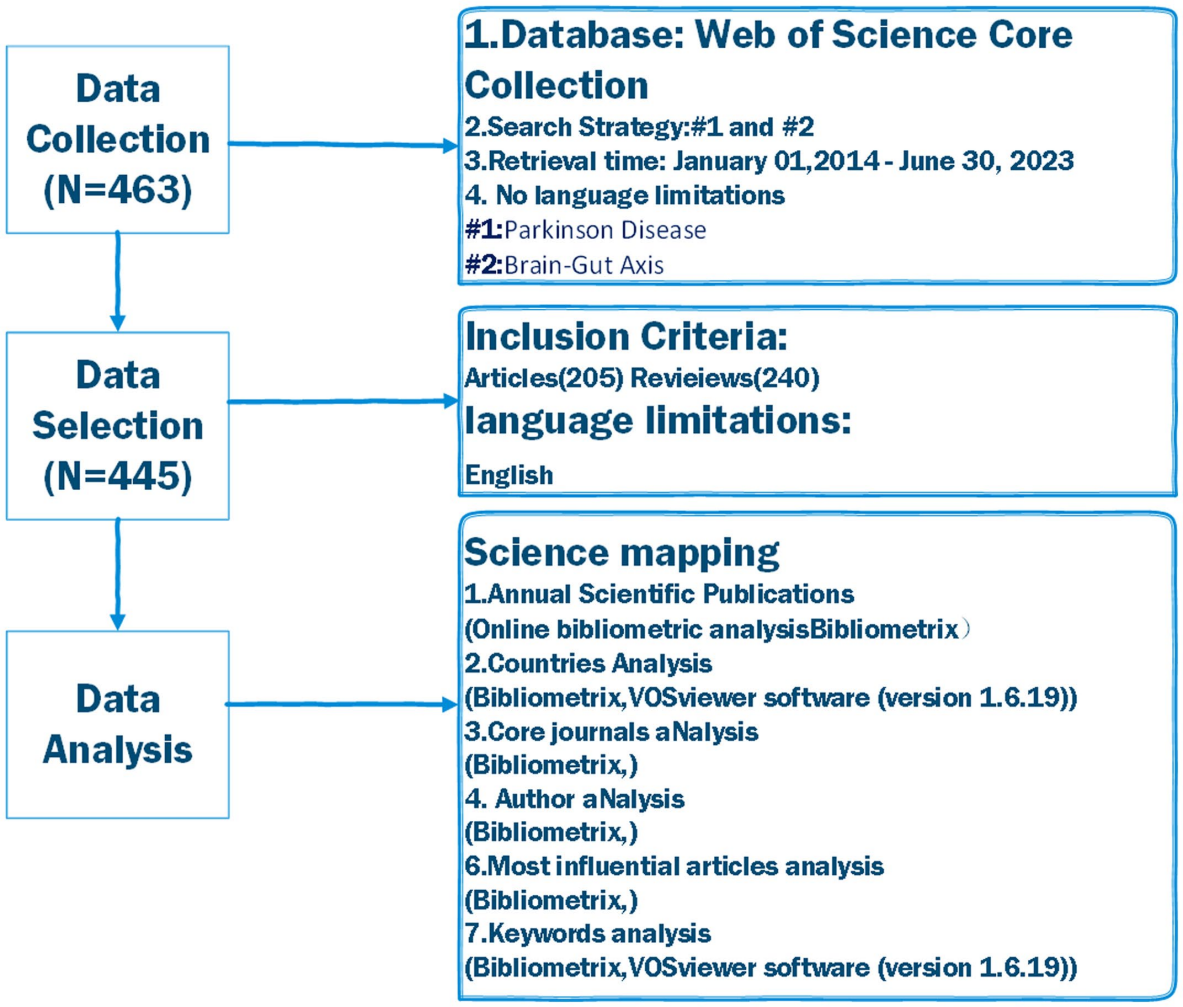
Flowchart of data collection and study design.

An online bibliometric analysis platform^1^ was used to analyze the annual number of publications. Country collaboration, core journals, authors, most influential articles, and keywords were analyzed using the Bibliometrix R package (12). Countries and keyword analysis were also performed using the VOSviewer software (version 1.6.19) (13). The workflow of the present study is shown in Figure 1.

## Results

### Brief bibliographic data

Table 1 summarizes the key quantitative characteristics of the final literature dataset employed in this study. Following the search strategy outlined in the methodology section, the dataset was extracted from the WoS database comprising scholarly articles published between 2014 and 2023. Consisting of 445 documents, the dataset exhibits an average citation count of 40.09 per article and an average publication-to-citation lag of 2.22 years. The research landscape of PD and the brain-gut axis is represented by 202 distinct journals, demonstrating a remarkable diversity of publication venues. Notably, the field has experienced significant growth, evident from the annual growth rate of 50.24%. Furthermore, the dataset contains extensive keyword information, including 978 author keywords and 1,258 additional keywords. Collaboration within the field has been prevalent, with an average of 6.34 authors per document and a notable proportion (23.6%) of international co-authorship, highlighting the collaborative nature of research on this topic.

**Table 1:** Main bibliographic information about the final dataset of the brain-gut axis and Parkinson disease.

Description	Results	Description	Results
Main information about data		Authors	
Timespan	2014:2023	Authors	2,354
Sources (Journals, Books, etc)	202	Authors of single-authored docs	20
Documents	445	Authors collaboration	
Annual Growth Rate %	50.24	Single-authored docs	21
Document Average Age	2.22	Co-authors per Doc	6.34
Average citations per doc	40.09	International co-authorships %	23.6
References	29,661		
Document contents			
Keywords plus (ID)	1,258		
Author’s keywords (DE)	978		

### Annual scientific publications

**Figure 2:**
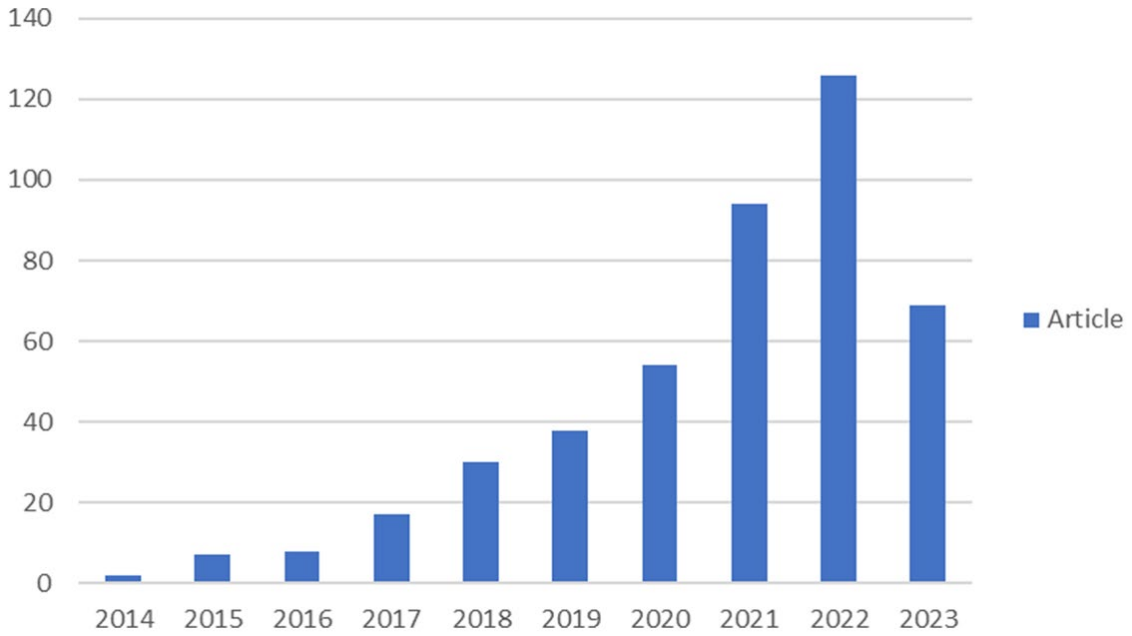
Trends in publications on the relationship between brain-gut axis and Parkinson disease using https://bibliometric.com.

A bibliometric analysis conducted between 2014 and 2023 yielded 445 publications on the brain-gut axis and PD (Figure 2). This period witnessed a significant increase in research output, potentially driven by the application of advanced “genomics” technologies and diverse strategies aimed at elucidating the pathophysiological underpinnings of PD, encompassing symptomatology, quality of life, and gastrointestinal dysfunction (14). This surge in publications underscores the active and growing research interest in the brain-gut axis and its potential role in PD pathogenesis.

### Country collaboration analysis

**Figure 3:**
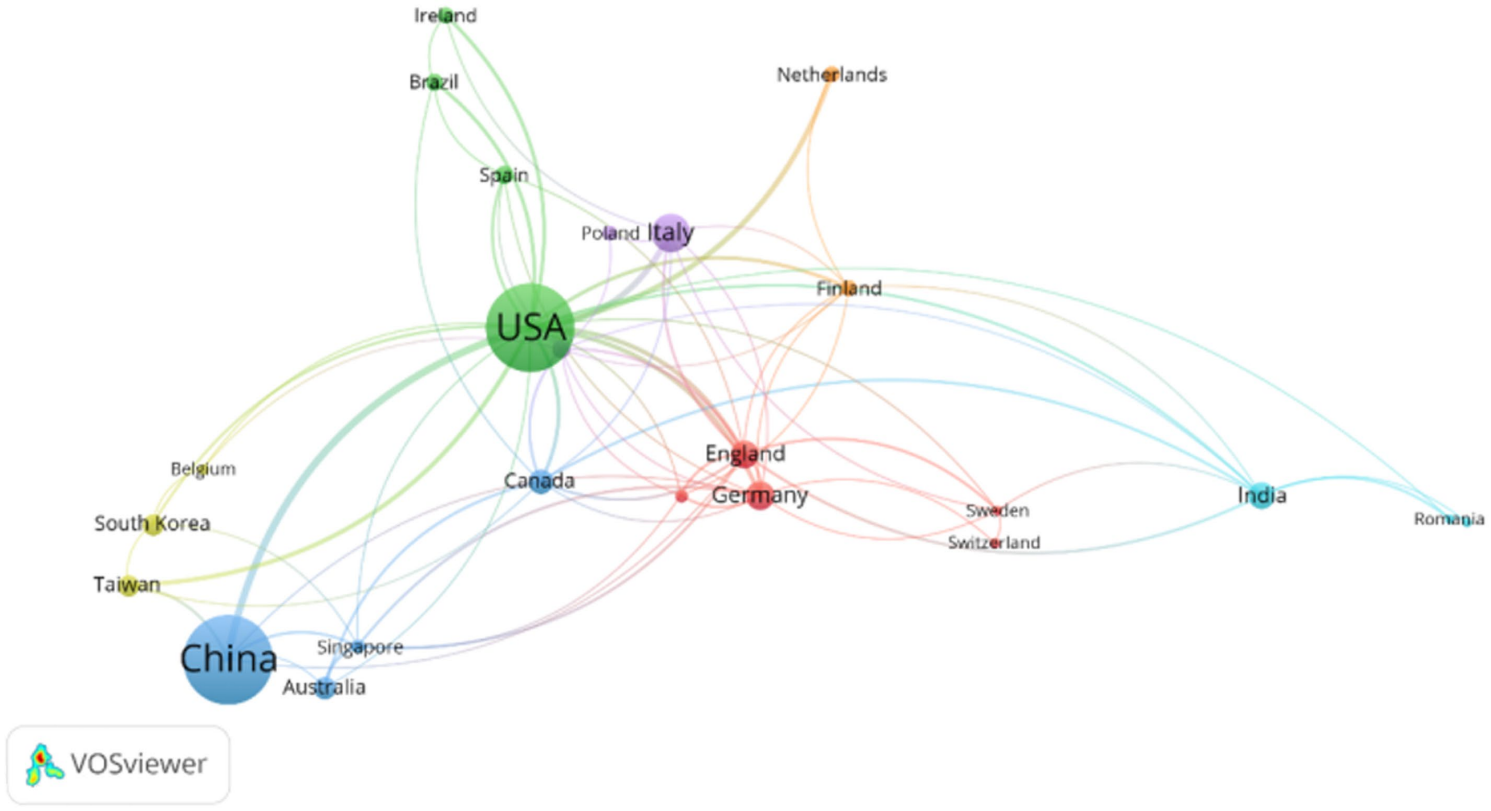
Map of international collaboration networks in research on brain-gut axis and Parkinson disease by using VOSviewer. Clusters are represented by dots of distinct colors, symbolizing varying levels of cooperation among countries with each cluster. China and the United States exhibited the highest number of publications and the closest collaboration.

**Figure 4:**
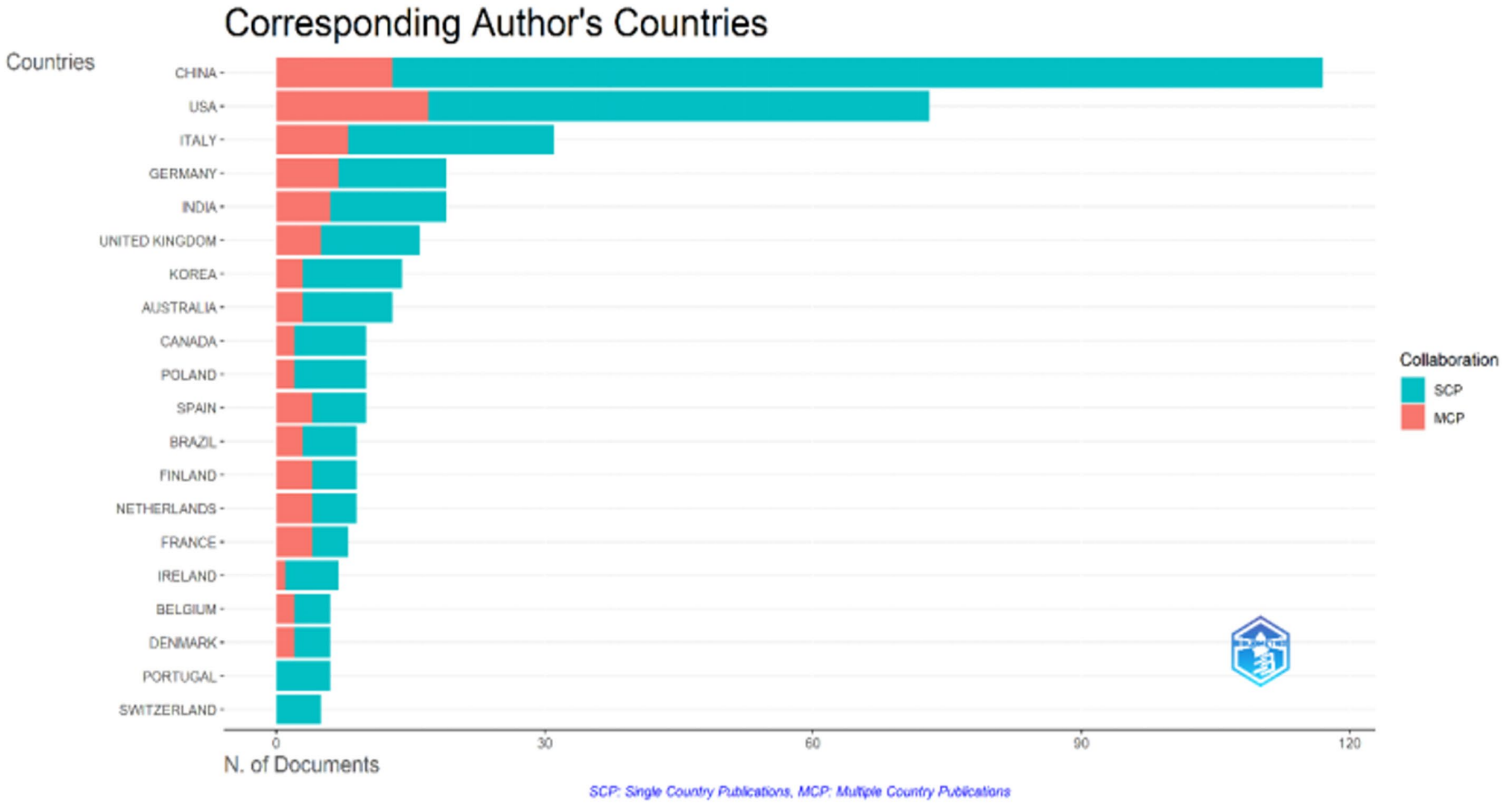
Geographical distribution of corresponding authors in the research area of brain-gut axis and Parkinson’s disease using bibliometrix.

Our analysis of corresponding author affiliation revealed that research on the brain-gut axis and PD spans a remarkable 44 countries or regions. As illustrated in Table 2, the top 10 most productive nations were China (117 publications, 26.3%), the United States (73 publications, 16.4%), Italy (31 publications, 7.0%), followed by Germany (19, 4.3%), India (19, 4.3%), the United Kingdom (16, 3.6%), Korea (14, 3.1%), Australia (13, 2.9%), Canada (10, 2.2%), and Poland (10, 2.2%). The United States ranked first in both total citations (TC = 3,832) and Multiple Country Publication (MCP = 17), underlining its significant role in this research field. This leadership is further evidenced by its collaborative network (Figure 3), showcasing frequent partnerships with various nations, including South Korea, India, and several European countries. Although ranking second overall, China exhibited the highest number of single-country publications (SCP = 104), suggesting a robust domestic research landscape. Notably, its MCP ratio (0.111) indicated a lower emphasis on international collaboration compared to the United States (Figure 4). Despite the individual strengths of these leading nations, the collaborative network analysis (Figure 3) underscores the critical importance of international cooperation in propelling scientific progress in this challenging field.

**Table 2:** Top 10 most productive countries (based on corresponding author’s countries) on brain-gut axis and Parkinson disease.

SCR	Country	NP	Percent %	TC	SCP	MCP	MCP_Ratio
1	China	117	26.3%	2,680	104	13	0.111
2	United States	73	16.4%	3,832	56	17	0.233
3	Italy	31	7.0%	1,201	23	8	0.258
4	Germany	19	4.3%	918	12	7	0.368
5	India	19	4.3%	443	13	6	0.316
6	United Kingdom	16	3.6%	436	11	5	0.313
7	Korea	14	3.1%	417	11	3	0.214
8	Australia	13	2.9%	111	10	3	0.231
9	Canada	10	2.2%	384	8	2	0.2
10	Poland	10	2.2%	539	8	2	0.2

### Core journals analysis

**Figure 5:**
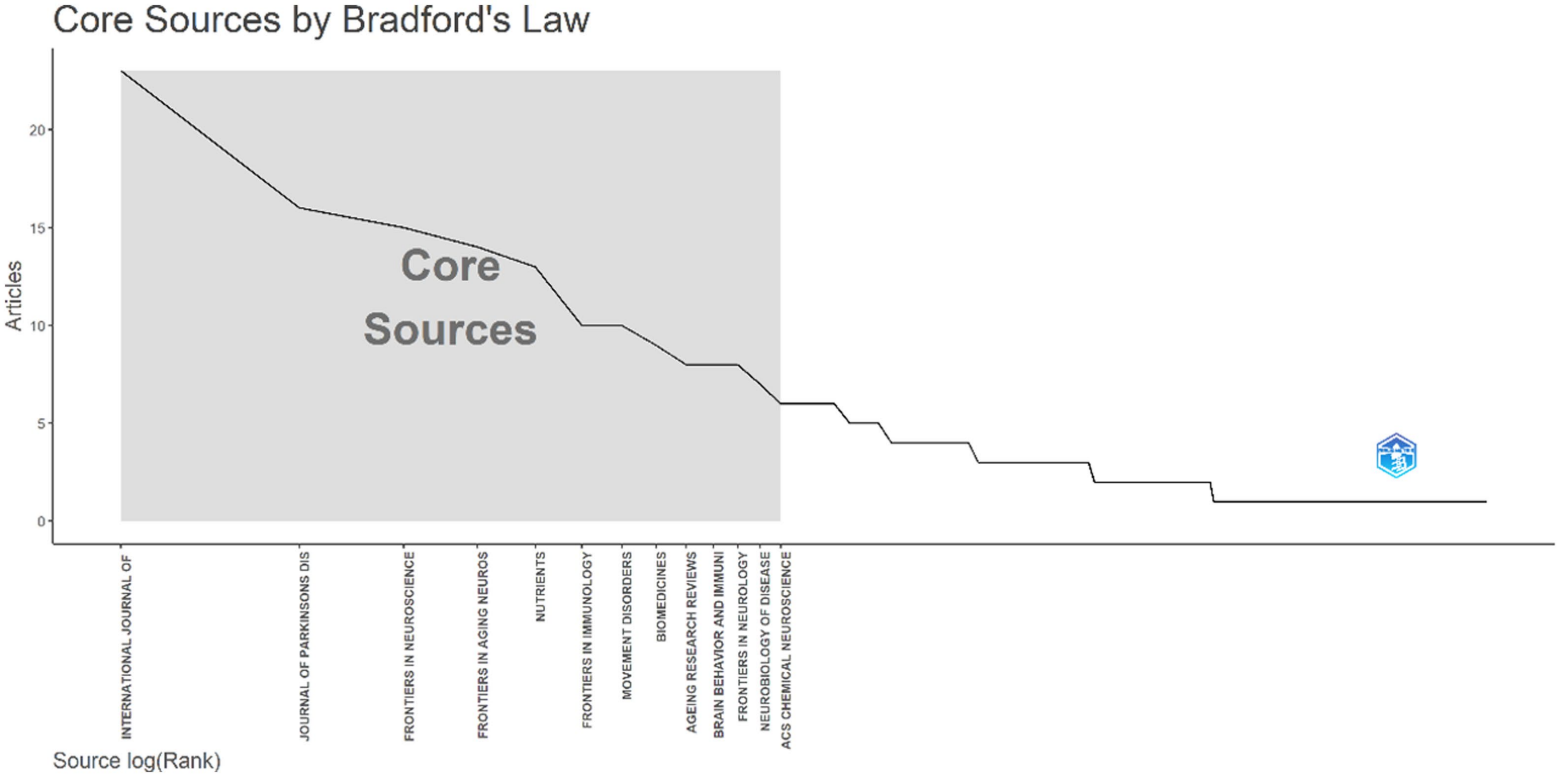
Distribution of core journals according to the principles of Bradford’s law using bibliometrix.

A comprehensive examination of the identified literature sources was conducted to assess the contribution of various journals to the field of PD and Brain-Gut Axis research. As shown in Table 1, a total of 445 publications pertaining to this specific research domain were published across 202 diverse journals between the years 2014 and 2023. Further analysis, as presented in Table 3, identified the top 10 most prolific journals alongside their corresponding cumulative citation counts and *h*-index values. Notably, the journals showcased in both Table 3 and Figure 5 collectively contributed 126 articles, representing a significant 28.31% of the entire dataset encompassing 445 publications. Among these, the International Journal of Molecular Sciences emerged as the leading contributor, publishing 23 articles, accounting for roughly 5% of the total publications within the analyzed timeframe. The Journal of Parkinson’s Disease (16 articles), Frontiers in Neuroscience (15 articles), and Frontiers in Aging Neuroscience (14 articles) closely followed this leading journal. Additionally, noteworthy contributions can be attributed to Nutrients, Frontiers in Immunology, Movement Disorders, Biomedicines, Aging Research Review, and Brain Behavior and Immunity, publishing 13, 10, 10, 9, 8, and 8 articles, respectively. It is further significant to mention that the International Journal of Molecular Sciences also secured the top position in terms of *h*-index (*h*-index = 10), signifying its high research impact. Meanwhile, Movement Disorders, boasting the longest publication history within the analyzed period (PY Start = 2,014), secured the top spot in terms of total citations (TC = 1,539).

**Table 3:** Top 10 most productive publication sources within the study area of the brain-gut axis in Parkinson disease (ranking in the table is based on this refers to the number of papers included in a specific journal).

Source journal	Rank	Articles	*h*_index	Total citations	PY_start
International Journal of Molecular Sciences	1	23	10	556	2018
Journal of Parkinson’s Disease	2	16	9	298	2015
Frontiers in Neuroscience	3	15	7	246	2018
Frontiers in Aging Neuroscience	4	14	7	254	2018
Nutrients	5	13	7	410	2018
Frontiers in Immunology	6	10	6	351	2019
Movement Disorders	7	10	8	1,539	2014
Biomedicines	8	9	4	82	2019
Aging Research Reviews	9	8	5	273	2018
Brain Behavior and Immunity	10	8	7	572	2018

### Author analysis

**Figure 6:**
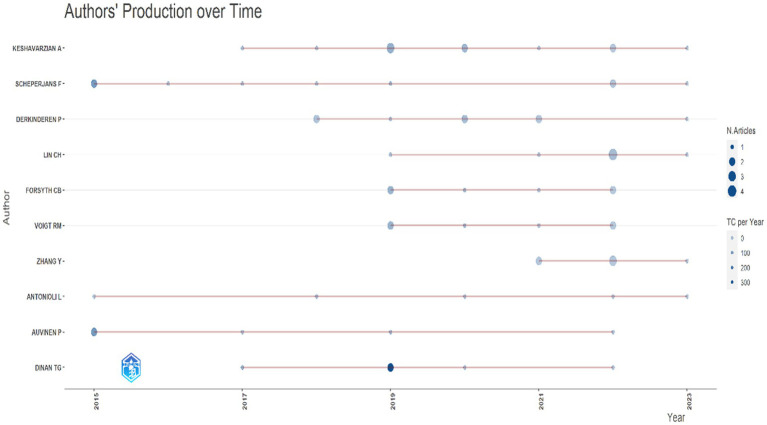
Author’s production over time by using bibliometrix.

Table 4 presents the *h*-index, total citations (TC), number of published papers (NP), and year of first publication (PY_start) for several key authors in the field of brain-gut axis and Parkinson’s Disease (PD). An integrated analysis of Table 4 and Figure 6 reveals that professors F. Scheperjans (TC = 1,491) and P. Auvinen (TC = 1,388) have demonstrated sustained research activity in this field since 2015. Notably, Professor Scheperjans possesses the highest *h*-index (*h* = 8) among the listed authors. Professors T.G. Dinan (TC = 2,131) and J.F. Cryan (TC = 2,110) exhibited the highest total citations, both commencing their publication records in 2017. In terms of published papers, Professor A. Keshavarzian, also starting in 2017, holds the distinction of boasting the most extensive output (NP = 11). These findings underscore the significant contributions of these authors and their impact on the understanding of the microbiota-gut-brain axis in the context of neurodegenerative diseases such as PD.

**Table 4:** Top 10 most prolific authors of the brain-gut axis in Parkinson disease.

Author	h_index	TC	NP	PY_start
Scheperjans F.	8	1,491	9	2015
Derkinderen P.	7	254	8	2018
Keshavarzian A.	7	692	11	2017
Lin C.H.	6	136	7	2019
Auvinen P.	5	1,388	5	2015
Dinan T.G.	5	2,131	5	2017
Cryan J.F.	4	2,110	4	2017
Dodiya H.B.	4	510	4	2017
Engen P.A.	4	411	5	2019
Forsyth C.B.	4	413	6	2019

### Most influential articles analysis

Table 5 presents the 10 most globally cited documents within the brain-gut axis and Parkinson’s disease research domain. It further provides detailed information on each document. Local citations assess the impact of a paper within a specific dataset based on the number of times it is cited within that dataset, while global citations represent a paper’s broader impact by measuring the total number of citations across all documents in a bibliographic database (15). The most frequently cited document within the analyzed dataset (“Gut microbiotas are related to Parkinson’s disease and clinical phenotype”) (16) received 189 local citations. This study suggests a link between altered intestinal microbiome composition and PD, specifically noting a significant reduction in the abundance of Prevotellaceae bacteria in PD patients. Additionally, this alteration was associated with both the severity of non-motor symptoms (e.g., constipation) and motor symptoms (e.g., postural instability and gait difficulty). In contrast, the most globally cited document (“The Microbiota-Gut-Brain Axis”) (17) received 1,518 citations, highlighting its wider impact across the scientific community. This article explored the crucial role of the microbiota-gut-brain axis in maintaining homeostasis and its influence on various neurological disorders, including psychiatric, neurodevelopmental, age-related, and neurodegenerative conditions. It further underscored the impact of various factors on the composition of the gut microbiota and emphasized the need for future research to elucidate underlying mechanisms and explore microbial-based interventions and therapeutic strategies for neuropsychiatric disorders.

**Table 5:** Top 10 most cited documents within the domain of the brain-gut axis and Parkinson disease.

Title	Year	First author	Journal	Local citations	Global citations	LC/GC ratio (%)	Normalized local citations	Normalized global citations
Gut microbiota are related to Parkinson’s disease and clinical phenotype	2015	Scheperjans F.	Movement Disorders	189	1,073	17.61	3.67	2.78
Functional implications of microbial and viral gut metagenome changes in early stage L-DOPA-naïve Parkinson’s disease patients	2017	Bedarf J.R.	Genome Medicine	86	332	25.9	4.12	1.89
Brain-gut-microbiota axis in Parkinson’s disease	2015	Mulak A.	World Journal of Gastroenterology	76	338	22.49	1.48	0.88
Neuroprotective effects of fecal microbiota transplantation on MPTP-induced Parkinson’s disease mice: Gut microbiota, glial reaction and TLR4/TNF-α signaling pathway	2018	Sun M.F.	Brain, Behavior, and Immunity	74	314	23.57	7.63	4.8
The gut-brain axis: is intestinal inflammation a silent driver of Parkinson’s disease pathogenesis?	2017	Houser M.C.	npj Parkinson’s Disease	65	315	20.63	3.11	1.79
Role of TLR4 in the gut-brain axis in Parkinson’s disease: a translational study from men to mice	2019	Perez-Pardo P.	Gut	59	207	28.5	3.94	1.84
Structural changes of gut microbiota in Parkinson’s disease and its correlation with clinical features	2017	Li W.	Science China Life Sciences	57	215	26.51	2.73	1.22
The microbiota-gut-brain axis	2019	Cryan J.F.	Physiological Reviews	48	1,518	3.16	3.21	13.48
Pathogenesis of Parkinson disease—the gut–brain axis and environmental factors	2015	Klingelhoefer L.	Nature Reviews Neurology	47	365	12.88	0.91	0.95
Unraveling gut microbiota in Parkinson’s disease and atypical parkinsonism	2019	Barichella M.	Movement Disorders	46	175	26.29	3.07	1.55

### Keywords analysis

**Figure 7:**
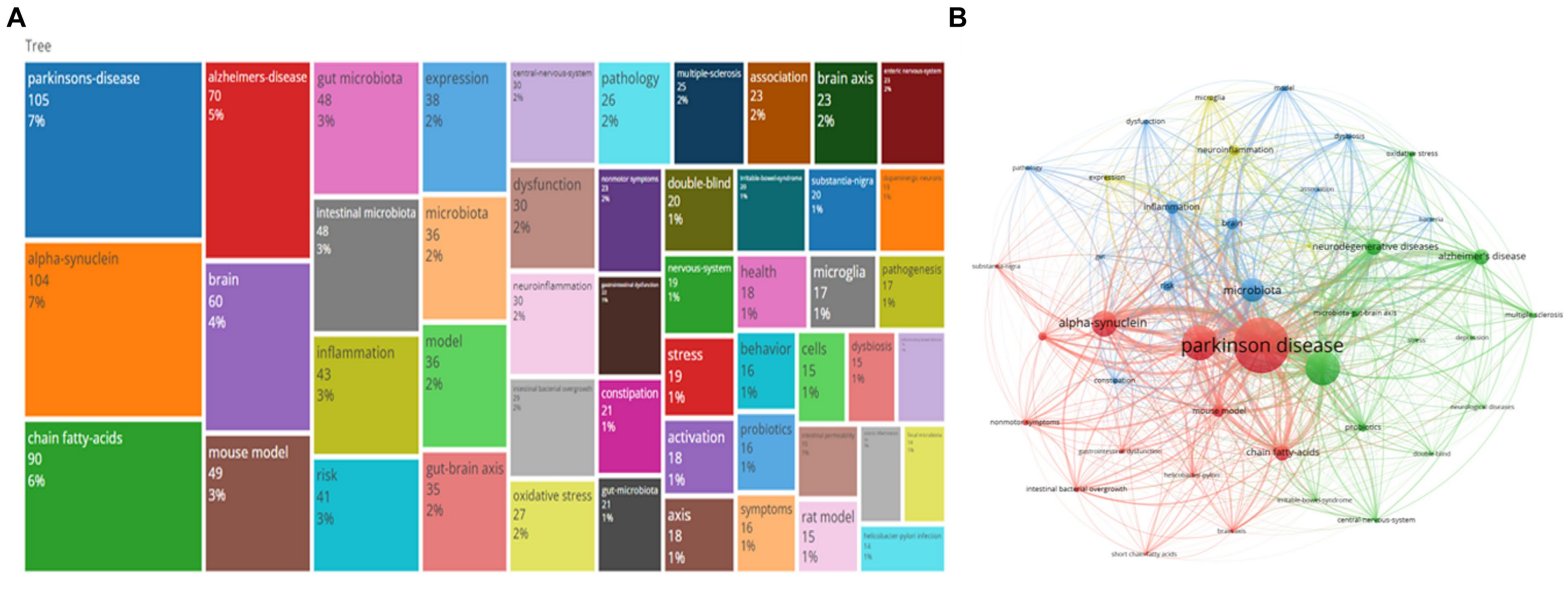
Analysis of research hotspots on the brain-gut axis and Parkinson, including **(A)** a tree visualization map of keywords using bibliometrix. The size of the differently colored squares indicates the frequency of occurrence. **(B)** A network visualization map of co-occurring keywords by using VOSviewer. Keywords in different fields are represented by dots of various colors. The size of the color indicates the frequency of occurrence.

Next, we analyzed the keywords in the above documents to reveal the knowledge structure regarding the brain-gut axis and Parkinson’s disease. Figure 7A displays the top 50 most frequently used search terms in this field. “Parkinson’s disease,” “alpha-synuclein,” and “short-chain fatty acids” emerged as the top three most popular terms, occurring 105, 104, and 90 times respectively, representing 7, 7, and 6% of the total keyword mentions. Following the merging of similar keywords (VOSviewer’s co-occurrence analysis using the full counting method, Unit of analysis: All keywords), Figure 7B presents the remaining 42 keywords for analysis. The size of each node corresponds to the keyword’s frequency, while the colors reflect the degree of correlation between them. Four distinct clusters emerged, grouping all terms.

The red cluster encompasses 13 keywords, including “alpha-synuclein,” “brain-gut axis,” “gastrointestinal dysfunction,” “short-chain fatty acids,” “Parkinson’s disease,” “mouse model,” and others. This cluster pertains to the “path mechanisms research” within the domain of Parkinson’s brain-gut axis research. Importantly, researchers explored the pathogenesis and pathological changes associated with Parkinson’s disease by examining factors such as alpha-synuclein aggregation, gut microbiota imbalance, intestinal inflammation, and intestinal dysfunction. Additionally, they utilized animal and cellular models to simulate the disease’s pathological process to gain further insights into its development and treatment. The green cluster includes 13 keywords such as “gut microbiota,” “neurodegenerative diseases,” “oxidative stress,” and “probiotics.” This cluster pertains to the “relationship between gut flora and Parkinson’s disease” within brain-gut axis Parkinson’s Research. Researchers in this field have investigated the impact of gut flora on the pathogenesis and pathological changes of Parkinson’s disease by studying factors such as gut flora imbalance, increased intestinal permeability, and intestinal inflammatory response. They further explored the link between gut flora and non-motor symptoms of Parkinson’s disease, as well as the role of gut flora in controlling the absorption and metabolism of Parkinson’s disease medications. The blue cluster contains 12 keywords like “dysbiosis,” “inflammation,” “microbiota,” “pathology,” and “risk.” This cluster aligns with the “correlational research” domain within Parkinson’s brain-gut axis research. Researchers employed statistical and epidemiological methods to explore correlations between factors such as gut flora, gut function, and neurological health, along with their associations with the risk of developing Parkinson’s disease. The yellow cluster consists of four keywords: “expression,” “inflammatory bowel disease,” “microglia,” and “neuroinflammation.” This cluster belongs to the “Gene Expression, Inflammatory Bowel Disease, Microglia, and Neuroinflammation” domain within Parkinson’s research. In this area, researchers investigated the connection between intestinal flora and the pathogenesis and pathology of Parkinson’s disease by examining elements such as abnormal gene expression, the link between inflammatory bowel disease and the risk of developing Parkinson’s disease, and the role of microglia in neuroinflammation.

**Figure 8:**
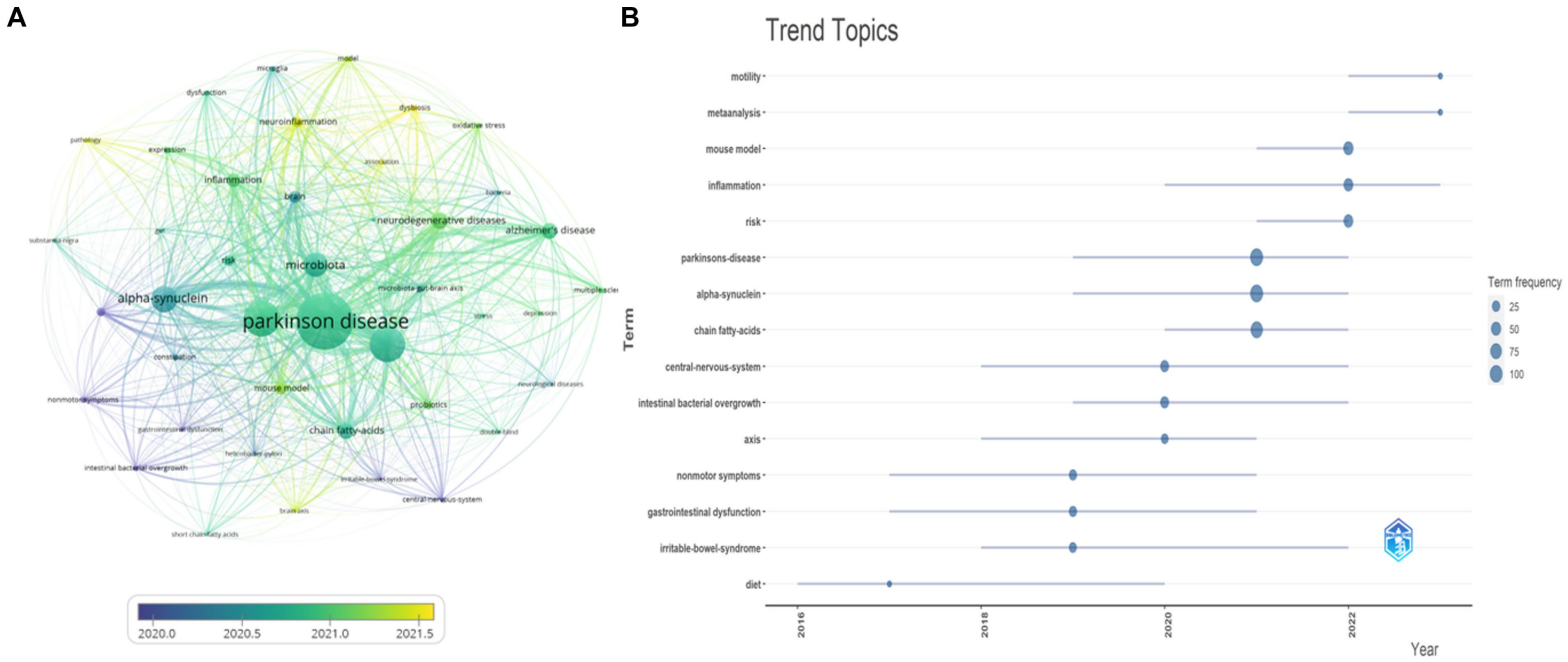
Analysis of current research trends on the brain-gut axis and Parkinson, including **(A)** an overlay visualization map of keywords using VOSviewer. The color gradient from blue to yellow indicates their chronological order of occurrence. The closer the color is to yellow, the more recent the research is for that particular keyword. **(B)** Trend topics evolving over time based on words presented using bibliometrix.

As shown in Figures 8A,B, based on keyword frequency and year of appearance, topics such as “Parkinson’s disease” and “alpha-synuclein” remain prominent in this field. In recent years, “mouse model,” “inflammation,” and “risk” have emerged as research hotspots, while topics like “central nervous system” and “intestinal bacterial overgrowth” have gained attention. Notably, “inflammation” and “dysbiosis” may represent the latest research trends. These findings offer valuable directions for future research in this field.

## Discussion

In the present study, we analyzed 445 publications on the brain-gut axis and Parkinson’s disease indexed in the Web of Science core database to map the recent research landscape using a bibliometric approach. The dataset encompassed articles published between January 1, 2014, and June 30, 2023. We observed an upward trend in the number of annual scientific publications on this topic, with an annual growth rate of 50.24%. This finding, further substantiated by the bibliometric evaluation, revealed a growing interest in the field of Parkinson’s disease and the brain-gut axis. The analysis of global cooperation networks identified the United States and China as leading nations, with the United States holding a significant advantage in academic influence. Furthermore, we observed significant collaborative relationships between the United States and other countries, including South Korea, India, and several European nations.

Table 3 lists the top 10 core journals in terms of journals and articles. Each of these journals published at least eight papers and had an H-index of at least 4. The H-index is a research performance indicator that incorporates the number and impact of publications into a single score (18). For example, the H-index of 10 for the journal “International Journal of Molecular Sciences” indicates that it has published 10 papers with at least 10 citations each. Total citations can serve as an important indicator of interest in a particular research area (19). Among these journals, “Movement Disorders” has the most total citations. It is a peer-reviewed medical journal covering all aspects of Parkinson’s disease and other movement disorders. The journal publishes original research articles and reviews related to the clinical aspects of these disorders, including Parkinson’s disease, dystonia, tremor, and other neurodegenerative disorders.^2^ “Delayed Gastric Emptying in Parkinson’s Disease” (20) is one of the early articles published in this journal. Additionally, it published the highly cited article “Gut microbiotas are related to Parkinson’s disease and clinical phenotype” (16).

Analysis of prolific authors revealed that Professor Scheperjans F has the highest h-index. His research focuses on neurology and psychiatry. His study found that the intestinal flora of PD patients differed significantly from the control group, with the abundance of Prevotellaceae reduced by 77.6%. This investigation, which explored the link between gut microbes and PD, provided important clues to understanding the cause of the illness (16). Professors T.G. Dinan and J.F. Cryan had the highest total number of citations in our analysis. Professor Dinan, a Professor of Psychiatry, focused his research on the immunological and endocrine components of irritable bowel syndrome and depression. His studies suggested that lifestyle choices and microbial metabolites may influence Parkinson’s disease risk and progression, and that the gut-brain axis and gut microbiota play crucial roles in neurodevelopmental, age-related, and neurodegenerative illnesses (21). More specifically, his research indicated that lifestyle variables, microbial metabolites, and the gut-brain axis may significantly impact the risk and progression of Parkinson’s disease, as well as other age-related, neurodegenerative, and neurodevelopmental disorders (17). Among others, Professor P. Auvinen’s research focused on genomics and DNA sequencing in biotechnology. Research has shown that decreased butyrate levels produced by bacteria may result in epigenetic changes in leukocytes and neurons, leading to more severe depressive symptoms in patients with Parkinson’s disease. Interestingly, Parkinson’s disease shares butyrate-dependent epigenetic changes with certain digestive and psychiatric disorders, suggesting a possible epidemiological connection (22). Professor A. Keshavarzian, whose research areas include Internal Medicine, Division of Digestive Diseases and Nutrition, has focused on the role of the gut microbiota in the pathogenesis of Parkinson’s disease. He emphasized the need for further research and suggested a possible future evaluation of microbiota-oriented therapeutic interventions for patients with Parkinson’s disease (23). Both professors play crucial roles and lead the field in studying the brain-gut axis and neurodegenerative diseases such as Parkinson’s disease.

Four primary clusters emerged from the co-occurrence network analysis of the keywords: biology and physiology, neurodegenerative diseases, diseases and symptoms, and experimental models. These clusters further included microbiota and their role in neurodegenerative diseases, and inflammation. Analyzing keyword frequency and year of appearance revealed the importance of topics like “Parkinson’s disease” and “alpha-synuclein” within this field. In recent years, “mouse model,” “inflammation,” and “risk” have emerged as hot research areas, while topics like “central nervous system” and “intestinal bacterial overgrowth” have gained attention. “Inflammation” and “dysbiosis” might be the latest research trends, suggesting potential directions for future investigation. Notably, the clustering analysis also highlighted the crucial role of the gut microbiome in both the development and progression of these diseases (17). Through genomic, metabolomic, and transcriptomic approaches, researchers have found that gut flora is closely associated with neurological processes, modulates behavior, and cognition, and is increasingly recognized as being associated with susceptibility to and progression of neurodegenerative and neuropsychiatric disorders (14). Dysbiosis significantly impacts gut-brain interactions during PD pathology, leading to alterations in bacterial metabolite activity (7). Future research on the gut-PD connection holds exciting prospects. This includes (1) deeper exploration of both functional and metabolic activities within gut flora through in-depth studies, (2) investigating the intricate connections between gut flora and the genetic, intestinal, and immune aspects of PD, (3) unraveling the potential role of gut flora in existing PD therapies, and (4) developing novel therapeutic approaches specifically targeting gut bacteria (14). While these studies offer a promising theoretical basis for developing new therapies by elucidating the role of gut flora in PD pathogenesis, limitations exist. Our analysis, restricted to WOSCC database publications (known for standardization and coverage but excluding non-indexed articles) (24), and English language (limiting its scope), spans January 1, 2014, to June 30, 2023, potentially missing recent trends due to publication delays resulting in insufficient time to accumulate publication and citation counts (23). Despite these limitations, we believe this work contributes to the field and hope to publish more comprehensive analyses in the future.

## Conclusion

This study offers valuable insights into the progress of brain-gut axis research related to Parkinson’s disease applications in the biomedical field. Moving forward, exploring the intricate connections between in-depth studies of gut flora’s functional and metabolic activities and the characteristics of Parkinson’s disease is crucial. Notably, transitioning from observational to interventional study designs presents a promising and developing trend, holding potential for personalized interventions and disease prediction.

## Data availability statement

The original contributions presented in the study are included in the article/Supplementary material, further inquiries can be directed to the corresponding author.

## Author contributions

LC: Writing – original draft, Writing – review & editing, Conceptualization, Data curation, Formal analysis, Software, Visualization, Project administration. JC: Investigation, Methodology, Supervision, Writing – review & editing. MW: Validation, Visualization, Writing – review & editing. PY: Resources, Writing – review & editing. XZ: Conceptualization, Writing – review & editing.

## Footnotes

^1^ https://bibliometric.com/

^2^ https://www.movementdisorders.org/
