# Supplementary material for: Analyzing the bibliometrics of brain-gut axis and Parkinson’s disease
Source: Front Neurol. 2024 Mar 7;15:1343303. doi: 10.3389/fneur.2024.1343303 (PMC10954898; doi:10.3389/fneur.2024.1343303)
Supplement: Supplementary file 1 [file Data_Sheet_1.docx]

Supplement Document 1: Mesh Terms and Search Strategy

Literature data were collected from the Science Citation Index Expanded (SCIE). To ensure comprehensiveness, the search subject term was identified through MESH Terms as search #1 AND #2

#1 ((TI=("Parkinson Disease" OR "Idiopathic Parkinson's Disease" OR "Lewy Body Parkinson's Disease" OR "Parkinson's Disease, Idiopathic" OR "Parkinson's Disease, Lewy Body" OR "Parkinson Disease, Idiopathic" OR "Parkinson's Disease" OR "Idiopathic Parkinson Disease" OR "Lewy Body Parkinson Disease" OR "Primary Parkinsonism" OR "Parkinsonism, Primary" OR "Paralysis Agitans")) OR AB=("Parkinson Disease" OR "Idiopathic Parkinson's Disease" OR "Lewy Body Parkinson's Disease" OR "Parkinson's Disease, Idiopathic" OR "Parkinson's Disease, Lewy Body" OR "Parkinson Disease, Idiopathic" OR "Parkinson's Disease" OR "Idiopathic Parkinson Disease" OR "Lewy Body Parkinson Disease" OR "Primary Parkinsonism" OR "Parkinsonism, Primary" OR "Paralysis Agitans")) OR AK=("Parkinson Disease" OR "Idiopathic Parkinson's Disease" OR "Lewy Body Parkinson's Disease" OR "Parkinson's Disease, Idiopathic" OR "Parkinson's Disease, Lewy Body" OR "Parkinson Disease, Idiopathic" OR "Parkinson's Disease" OR "Idiopathic Parkinson Disease" OR "Lewy Body Parkinson Disease" OR "Primary Parkinsonism" OR "Parkinsonism, Primary" OR "Paralysis Agitans")

#2((TI=("Brain-Gut Axis" OR "Axis, Brain-Gut" OR "Brain Gut Axis" OR "Gut and Brain Axis" OR "Gut-Brain Axis" OR "Axis, Gut-Brain" OR "Gut Brain Axis" OR "Brain and Gut Axis" OR "Microbiota-Gut-Brain Axis" OR "Axis, Microbiota-Gut-Brain" OR "Microbiota Gut Brain Axis" OR "Brain-Gut-Microbiome Axis" OR "Axis, Brain-Gut-Microbiome" OR "Brain Gut Microbiome Axis" OR "Microbiome-Gut-Brain Axis" OR "Axis, Microbiome-Gut-Brain" OR "Microbiome Gut Brain Axis" OR "Gut-Brain-Microbiome Axis" OR "Axis, Gut-Brain-Microbiome" OR "Gut Brain Microbiome Axis" OR "Microbiome-Brain-Gut Axis" OR "Axis, Microbiome-Brain-Gut" OR "Microbiome Brain Gut Axis" OR "Microbiota-Brain-Gut Axis" OR "Axis, Microbiota-Brain-Gut" OR "Microbiota Brain Gut Axis")) OR AB=("Brain-Gut Axis" OR "Axis, Brain-Gut" OR "Brain Gut Axis" OR "Gut and Brain Axis" OR "Gut-Brain Axis" OR "Axis, Gut-Brain" OR "Gut Brain Axis" OR "Brain and Gut Axis" OR "Microbiota-Gut-Brain Axis" OR "Axis, Microbiota-Gut-Brain" OR "Microbiota Gut Brain Axis" OR "Brain-Gut-Microbiome Axis" OR "Axis, Brain-Gut-Microbiome" OR "Brain Gut Microbiome Axis" OR "Microbiome-Gut-Brain Axis" OR "Axis, Microbiome-Gut-Brain" OR "Microbiome Gut Brain Axis" OR "Gut-Brain-Microbiome Axis" OR "Axis, Gut-Brain-Microbiome" OR "Gut Brain Microbiome Axis" OR "Microbiome-Brain-Gut Axis" OR "Axis, Microbiome-Brain-Gut" OR "Microbiome Brain Gut Axis" OR "Microbiota-Brain-Gut Axis" OR "Axis, Microbiota-Brain-Gut" OR "Microbiota Brain Gut Axis")) OR AK=("Brain-Gut Axis" OR "Axis, Brain-Gut" OR "Brain Gut Axis" OR "Gut and Brain Axis" OR "Gut-Brain Axis" OR "Axis, Gut-Brain" OR "Gut Brain Axis" OR "Brain and Gut Axis" OR "Microbiota-Gut-Brain Axis" OR "Axis, Microbiota-Gut-Brain" OR "Microbiota Gut Brain Axis" OR "Brain-Gut-Microbiome Axis" OR "Axis, Brain-Gut-Microbiome" OR "Brain Gut Microbiome Axis" OR "Microbiome-Gut-Brain Axis" OR "Axis, Microbiome-Gut-Brain" OR "Microbiome Gut Brain Axis" OR "Gut-Brain-Microbiome Axis" OR "Axis, Gut-Brain-Microbiome" OR "Gut Brain Microbiome Axis" OR "Microbiome-Brain-Gut Axis" OR "Axis, Microbiome-Brain-Gut" OR "Microbiome Brain Gut Axis" OR "Microbiota-Brain-Gut Axis" OR "Axis, Microbiota-Brain-Gut" OR "Microbiota Brain Gut Axis")
