# Supplementary material for: Analyzing the bibliometrics of brain-gut axis and Parkinson’s disease
Source: Front Neurol. 2024 Mar 7;15:1343303. doi: 10.3389/fneur.2024.1343303 (PMC10954898; doi:10.3389/fneur.2024.1343303)
Supplement: Supplementary file 2 [file Presentation_1.PPTX]

## Slide 1
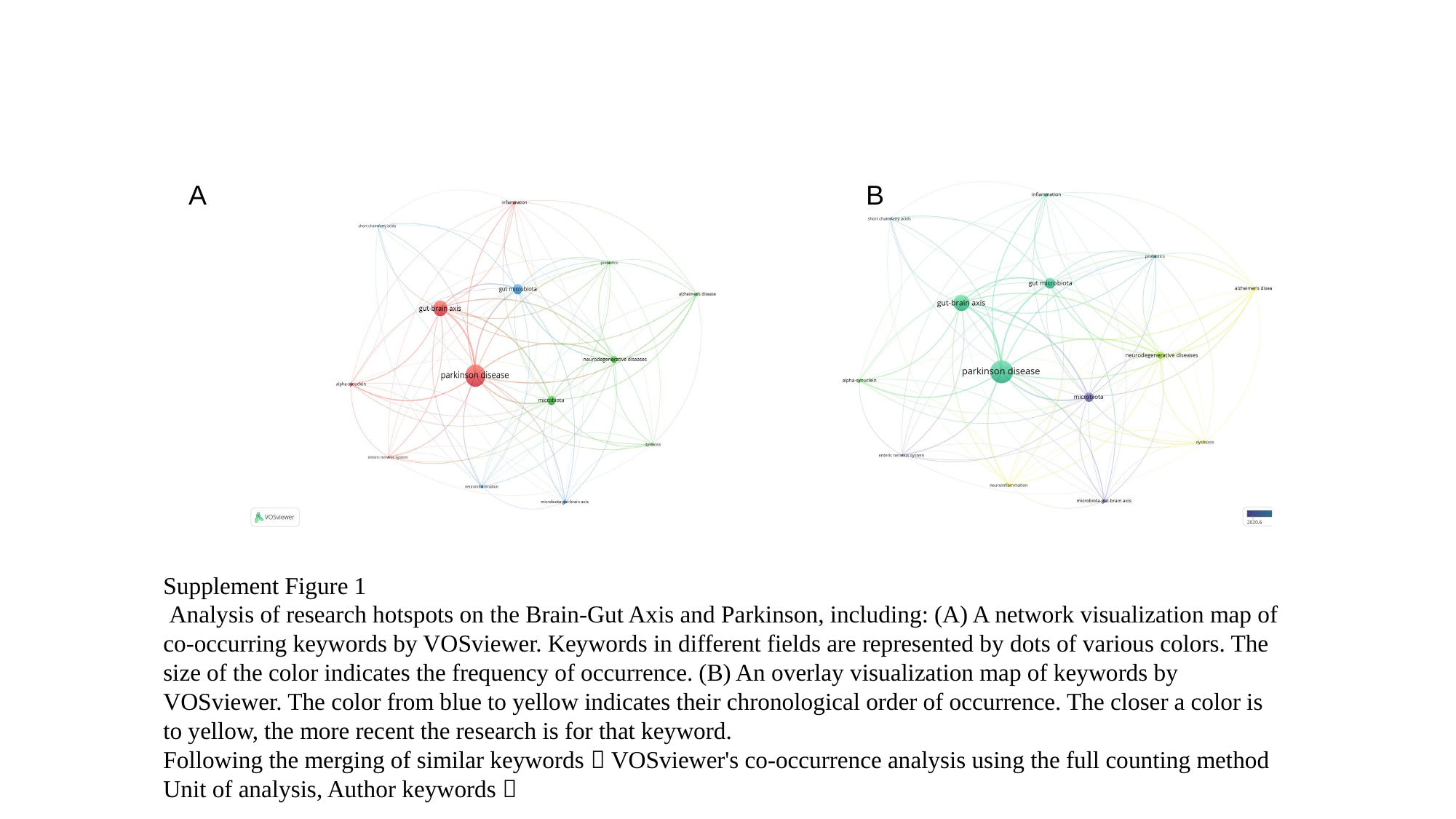

A
B
Supplement Figure 1
 Analysis of research hotspots on the Brain-Gut Axis and Parkinson, including: (A) A network visualization map of co-occurring keywords by VOSviewer. Keywords in different fields are represented by dots of various colors. The size of the color indicates the frequency of occurrence. (B) An overlay visualization map of keywords by VOSviewer. The color from blue to yellow indicates their chronological order of occurrence. The closer a color is to yellow, the more recent the research is for that keyword.
Following the merging of similar keywords（VOSviewer's co-occurrence analysis using the full counting method Unit of analysis, Author keywords）

## Slide 2
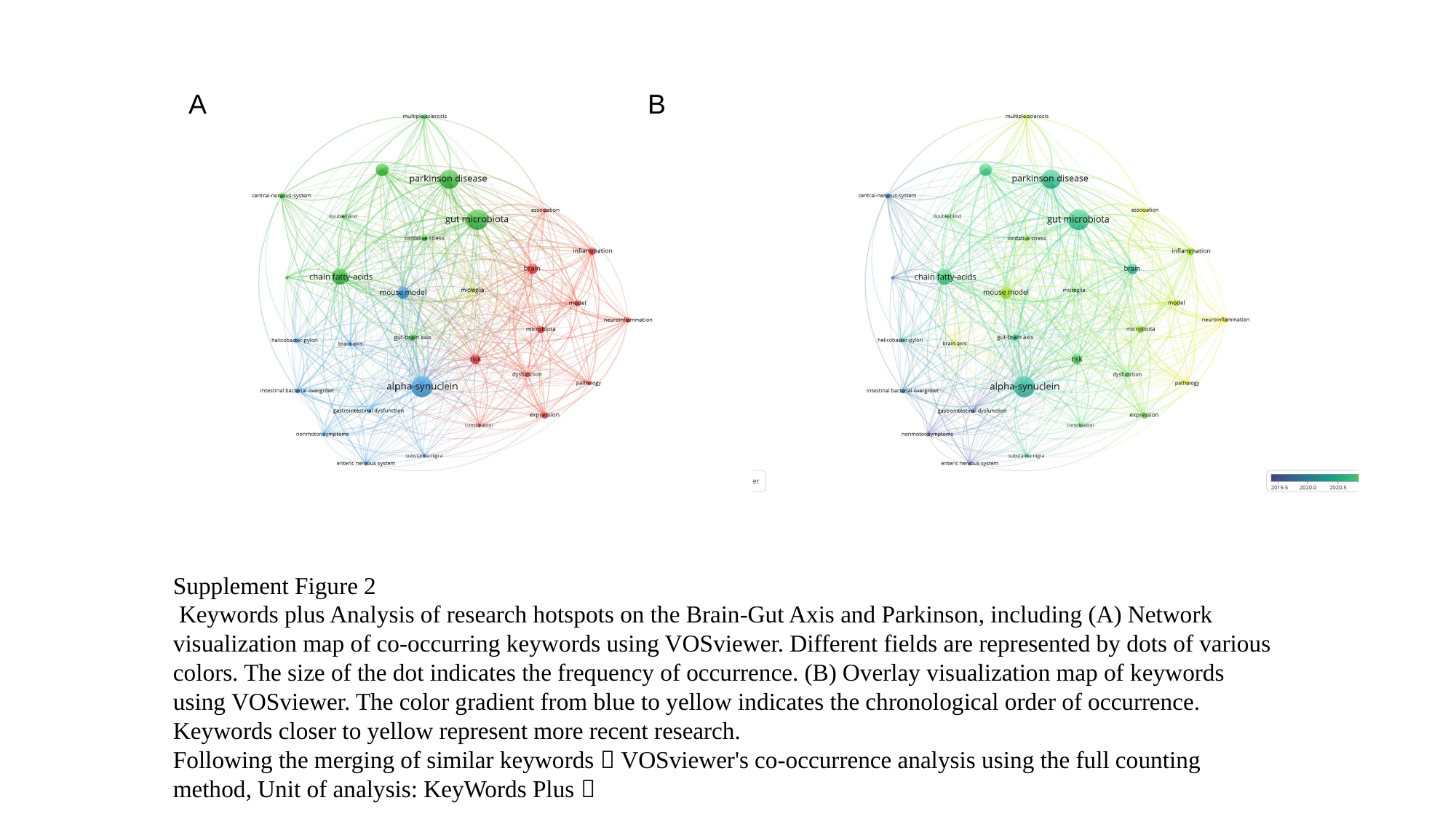

A
B
Supplement Figure 2
 Keywords plus Analysis of research hotspots on the Brain-Gut Axis and Parkinson, including (A) Network visualization map of co-occurring keywords using VOSviewer. Different fields are represented by dots of various colors. The size of the dot indicates the frequency of occurrence. (B) Overlay visualization map of keywords using VOSviewer. The color gradient from blue to yellow indicates the chronological order of occurrence. Keywords closer to yellow represent more recent research.
Following the merging of similar keywords（VOSviewer's co-occurrence analysis using the full counting method, Unit of analysis: KeyWords Plus）
